# A Stepwise Approach to 1,4,10,13‐Tetraaza‐18‐Crown‐6 Ether

**DOI:** 10.1002/open.70201

**Published:** 2026-05-05

**Authors:** Pieter Troosters, Lara Bruneel, Wim Dehaen, Tomas Opsomer

**Affiliations:** ^1^ Nuclear Energy Technology Belgian Nuclear Research Centre (SCK CEN) Mol Belgium; ^2^ Department of Chemistry KU Leuven Leuven Belgium; ^3^ Nuclear Medical Applications Belgian Nuclear Research Centre (SCK CEN) Mol Belgium

**Keywords:** crown compounds, macrocycles, macrocyclization, stepwise synthesis, tetraaza‐18‐crown‐6 ether

## Abstract

A two‐step approach to the synthesis of tetraaza‐18‐crown‐6 ether was evaluated to address the limitations of the currently used one‐step macrocyclization, such as low yield due to substantial side product formation. In this route, the reaction conditions were optimized, reducing cyclization to the undesired nine‐membered ring by a factor of eight. This resulted in a 50% yield for the two‐step macrocyclization, or 28% when column chromatography was avoided, both significantly higher than the 16% yield obtained when reproducing the one‐step method.

## Graphical abstract

A two‐step synthetic strategy to tetraaza‐18‐crown‐6 was developed to overcome the low yield of the conventional one‐step method. Optimized conditions significantly suppressed formation of the undesired nine‐membered ring. A three‐fold increase in yield of the macrocyclization step was obtained, or two‐fold when column chromatography was avoided.

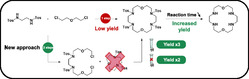

## 1 Introduction

**SCHEME 1:**
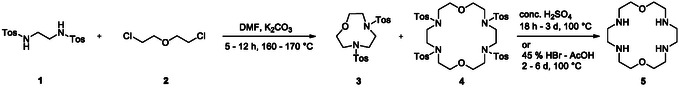
One‐step approach to TA18C6.

Aza‐crown ethers, such as tetraaza‐18‐crown‐6 ether **5** (TA18C6, Scheme 1), are interesting ligand scaffolds due to their ability to form stable complexes with various metal ions [1, 2, 3]. Whereas classical crown ethers are mainly known for their high affinity toward alkali metals, tetraaza‐crown ethers tend to form more stable complexes with soft transition metals such as Ag(I), Cu(I,II), Ni(II), Zn(II), and Cd(II) [2, 4, 5, 6]. Importantly, the nitrogen atoms in aza‐crown ethers can be functionalized, allowing to increase the denticity and to tailor the ligand's coordinating properties. This has enabled the development of strong complexing agents for lanthanides and actinides, which typically prefer high coordination numbers (≥8) [7]. In recent years, TA18C6 functionalized with four acetate groups, referred to as Crown, has been identified as a potent chelator for theranostic radiopharmaceuticals [3, 8, 9, 10, 11]. Crown forms stable complexes with actinium‐225 and with the terbium isotopes terbium‐155 and terbium‐161 under mild conditions, showing much faster labeling kinetics compared to the benchmark chelator DOTA [3, 8, 9, 10, 11]. To further investigate the potential of TA18C6, a more efficient and reproducible synthesis toward this ligand is required.

**SCHEME 2:**
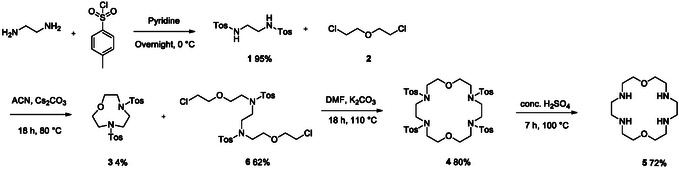
Stepwise approach to TA18C6.

Following the Richman–Atkins general procedure for the synthesis of macrocyclic polyamines and amino ethers, TA18C6 **5** has been prepared first by Biernat et al. via a one‐step cyclization reaction of ditosylethylenediamine **1** and bis(2‐chloroethyl) ether **2** (Scheme 1) [12, 13, 14]. This reaction was performed for 5 h at 170°C using K_2_CO_3_ as the base and DMF as the solvent. Under these conditions, the target compound **4** was obtained in a yield of 35%, while the undesired 9‐membered cyclization product, diaza‐9‐crown‐3 ether **3**, was also detected but not quantified [14]. Over the years, variations in reaction conditions have been employed, with temperatures ranging from 160°C to 170°C and reaction times between 5 and 12 h. Nonetheless, yields of 35% or lower for **4** were achieved without quantifying the yield of side product **3** [3, 15, 16, 17, 18, 19, 20]. Wade et al. claimed a yield of 100% for this reaction; however, the result appears questionable given that it remains an isolated case [4]. Craig et al. used an adapted method to synthesize compound **4** [6]. The reaction was performed for 12 h at room temperature, followed by 4 h at 60°C, using diethylene glycol ditosylate instead of bis(2‐chloroethyl) ether **2** with Cs_2_CO_3_ as the base. The desired product **4** was obtained in a 20% yield, while notably, the undesired side product **3** was isolated in a yield of 41%. In general, reproducing high yields for the one‐step cyclization step seemed to be challenging since in more recent publications, yields below 20% were mentioned [3, 8, 9, 11, 21]. Furthermore, the tosyl deprotection of compound **4** required additional attention. The deprotection has been performed in either a 45% HBr‐AcOH solution or concentrated H_2_SO_4_, resulting in low to fair yields of 10%–50% of TA18C6 **5** [2, 4, 14, 15, 16, 22]. The inconsistent results and low yields observed in both steps of the synthesis highlight the need for a robust and reproducible synthetic approach. In pursuit of this objective, minimizing the formation of the undesired 9‐membered cyclization product **3** will be key. Therefore, we investigated a stepwise strategy for the macrocyclization to improve overall efficiency (Scheme 2).

## 2 Results and Discussion

### 2.1 One‐Step Macrocyclization

To investigate the reproducibility of the literature procedures, the macrocyclization reaction reported by Biernat et al. and Yang et al. was repeated (Scheme 1) [3, 14]. First, ditosylethylenediamine **1** was prepared in 95% yield as previously described [3]. Next, the macrocyclization was carried out at 170°C for 5 and 12 h using K_2_CO_3_ and DMF. The reactions were performed with 5 g of starting material **1**, whereas the literature reports scales of 1.85 and 7.4 g for the 5‐ and 12‐h reactions, respectively. After reaction, compound **4** was precipitated with H_2_O from the DMF solution resulting in a sticky, impure product. By sonicating the sticky solid in acetone, the pure compound **4** could be obtained as a white solid in a yield of 16% for the 5‐h reaction. Next, the acetone fraction was evaporated and purified via column chromatography to obtain side product **3** in 31% yield for the 5‐h reaction. Similar yields were obtained after heating for 12 h, indicating that a reaction time of 5 h is sufficient. The yield of product **4** is markedly lower than the 35% reported by Biernat et al., but comparable to the yield obtained by Yang et al. [3, 14]. Additionally, the results seem to refute the 100% yield reported by Wade et al., as they reported identical reaction conditions, i.e., 5 h at 170°C [4]. The 5‐h reaction was also repeated with Cs_2_CO_3_ as the base, which resulted in yields of 13% and 26% for product **4** and side product **3**, respectively. We believe that the differences in yield are too small to draw any conclusion about the role of the alkali metals in the cyclization reaction.

### 2.2 Two‐Step Macrocyclization

The two‐step macrocyclization uses the same reagents as the original synthesis described in literature [14]. For the two‐step reaction, 100 mg of compound **1** was reacted with an excess of inexpensive bis(2‐chloroethyl) ether **2** under concentrated conditions to obtain the acyclic intermediate **6**, and to minimize the formation of side product **3** (Scheme 2). Different reaction conditions were evaluated to optimize this reaction step as visualized in Table 1. The reactions were monitored using TLC analysis, indicating a reaction time of 18 h was sufficient to obtain full conversion of starting material **1**. Cs_2_CO_3_ and ACN were identified as the optimal base and solvent for the reaction, respectively (Table 1, Entry 1–5). The higher solvation of Cs^+^ in polar aprotic solvents reduces anion‐cation association, which is expected to enhance the overall reactivity [23, 24]. A slightly higher yield of **6** was observed in ACN, whereas 5% of starting material **1** remained after 18 h when the reaction was performed in DMF. Next, the volume of ACN was optimized by raising the volume from 0.25 to 2 mL. A volume of 0.5 mL afforded the highest yield of the desired product (72%) and the lowest yield of side product **3** (4%) (Table 1, Entry 5). It could be concluded that more dilute conditions lead to an increased formation of side product **3**. However, if the amount of solvent is too low, the product yield decreases, presumably due to the reduced polarity of the reaction medium (Table 1, Entry 5–8). Subsequently, the effect of varying the molar equivalents of bis(2‐chloroethyl) ether **2** was evaluated.

**TABLE 1:** Different reaction conditions tested for the synthesis of acyclic intermediate **6** from ditosylethylenediamine **1** and bis(2‐chloroethyl) ether **2**.^a^

Entry	Base/equiv.	Solvent/V, mL	*T*, °C	*V* 2, mL/equiv.	Yield 6, %	Yield 3, %
**1** b	Cs_2_CO_3_/2.5	DMF/0.5	80	0.5/16	66	NQ
**2**	K_2_CO_3_/2.5	DMF/0.5	80	0.5/16	58	NQ
**3**	K_2_CO_3_/2.5	ACN/0.5	80	0.5/16	59	27
**4** c	Na_2_CO_3_/2.5	ACN/0.5	80	0.5/16	0	13
**5**	Cs_2_CO_3_/2.5	ACN/0.5	80	0.5/16	72	4
**6**	Cs_2_CO_3_/2.5	ACN/0.25	80	0.5/16	67	4
**7**	Cs_2_CO_3_/2.5	ACN/1.0	80	0.5/16	67	13
**8**	Cs_2_CO_3_/2.5	ACN/2.0	80	0.5/16	60	22
**9**	Cs_2_CO_3_/2.5	ACN/2.0	80	0.25/8	62	13
**10**	Cs_2_CO_3_/2.5	ACN/0.5	80	1.0/32	77	7
**11**	Cs_2_CO_3_/2.5	ACN/0.5	60	0.5/16	64	7
**12** d	Cs_2_CO_3_/2.5	ACN/0.5	80	0.55/16^d^	73	13

Abbreviation: NQ, not quantified.

a 0.27 mmol of **1** was used. The reaction time was 18 h.

b There was still 5% of starting material **1** present.

c There was still 64% of starting material **1** present.

d Bis(2‐bromoethyl) ether was used instead of bis(2‐chloroethyl) ether **2**.

Decreasing the amount of **2** from 16 to 8 equiv. lowered the yield from 72% to 62%, whereas increasing it to 32 equiv. resulted only in a small improvement to 77% (Table 1, Entry 9 and 10). These results confirmed that a large excess of **2** is needed. Decreasing the reaction temperature did not improve the reaction yield (Table 1, Entry 11). Finally, the conditions of entry 5 were repeated with bis(2‐bromoethyl) ether instead of bis(2‐chloroethyl) ether **2** (Table 1, Entry 12). Product **6** was obtained in similar yield, with a slight increase in the formation of side product **3**. The conditions from entry 5 were selected for the 5‐gram scale reaction, as they require only half the amount of **2** compared to entry 10, with a yield reduction of just 5%. On 5‐gram scale, the presence of **2** compromised the separation of product **6** via column chromatography. Therefore, product **6** was first coprecipitated with side product **3** using cyclohexane. This resulted in the formation of a brown oil and clear crystals, both mainly consisting of intermediate **6**, when leaving the mixture overnight in the fridge. The oil and crystals were not separated to minimize product loss, and the precipitates were washed with cyclohexane to remove the excess **2**. TLC analysis confirmed that some product loss in the cyclohexane fractions was unavoidable. Next, the product was purified via column chromatography resulting in a yield of 62% and 4% of side product **3**. Besides using this purified intermediate **6** (Procedure A), it was also possible to work further with the coprecipitated mixture of compound **6** and side product **3** (Procedure B). However, four consecutive washes with cyclohexane were needed to remove traces of bis(2‐chloroethyl) ether **2**, resulting in a loss of approximately 11 mol% of intermediate **6** (see calculations based on ^1^H NMR data in the Supporting Information).

The second step is the ring closure of **6** by reacting it with another equivalent of ditosylethylenediamine **1**. This reaction was performed in diluted conditions to avoid oligomerization. Product **4** was obtained as a white solid by precipitation with water from a DMF solution, when the reaction was performed with purified intermediate **6**. Three different carbonate salts were evaluated for this reaction. Using K_2_CO_3_ resulted in a high yield of 80% (Procedure A). Na_2_CO_3_, on the other hand, resulted in a yield of only 23%, with 54% of starting material **6** remaining. A similar decrease in reactivity when using Na_2_CO_3_ was also reported by Biernat et al. [14]. When Cs_2_CO_3_ was used, a lower yield of 61% was obtained despite full conversion of the starting material, likely due to a higher degree of oligomerization. A plausible explanation for the higher yield obtained with K_2_CO_3_ is the template effect of K^+^, whose ionic size fits optimally within 18‐crown‐6 ethers [14, 25].

Starting from the precipitated mixture of **3** and **6** (Procedure B), a sticky impure product was obtained after precipitation with water. Therefore, sonication of the residue in acetone was required to obtain the pure compound **4** as a white solid. The overall yield of procedure B was 28%, which is lower than the 50% obtained via procedure A. TLC analysis of both the H_2_O/DMF and acetone phases confirmed that no product was present in either phase. This suggests that the reduced yield is primarily due to a less efficient ring‐closure reaction when starting from a crude mixture of **6**, as well as product loss during the washes with cyclohexane. Nevertheless, the avoidance of column chromatography remains a notable advantage of Procedure B. In our hands, the overall yields of the two‐step macrocyclization are, respectively, two‐ and threefold improvements compared to the one‐step method.

### 2.3 Tosyl Deprotection

The final step of the synthesis involves deprotection of the tosyl groups to afford compound **5**. The tosyl groups were removed by heating in concentrated H_2_SO_4_, following a modified literature procedure [14, 16]. An important modification was the reduction in reaction time from 3 days mentioned in literature to 7 h in this work [14, 16], which increased the yield significantly from 50% to 72%.

### 2.4 Alternative Reagents

A different route starting from ditosyl‐2,2′‐oxybis(ethan‐1‐amine) **7,** [26] and 1,2‐dichloroethane (DCE) was also investigated (Scheme S1). However, attempts to purify **4** were unsuccessful due to the formation of side products, including β‐elimination products, along with incomplete conversion of the starting materials. The presence of β‐elimination products was confirmed via mass spectrometry. Incomplete conversion was also an issue in the preparation of the acyclic intermediate (Table S1). Therefore, further efforts on this route were discontinued.

## 3 Conclusion

In conclusion, an efficient and scalable synthetic procedure for the stepwise synthesis of TA18C6 **5** was established. The optimized two‐step cyclization approach effectively minimized the formation of the undesired side product **3**, as its yield was reduced by a factor of 8. The overall yield of the macrocyclization step was 50% when purifying intermediate **6** via column chromatography, and 28% when working with a coprecipitated mixture of **3** and **6**. These findings highlight that carrying out the macrocyclization in a more controlled manner leads to higher product yields and substantially reduces side product formation. We anticipate that this approach will facilitate improved access to TA18C6‐based chelators, which hold promise in targeted radionuclide therapy.

## 4 Experimental Section

### 4.1 Materials and Methods

All (dry) solvents were purchased from Thermo Fisher Scientific. All the reagents used for synthesis were purchased from Fluorochem, Sigma–Aldrich, Thermo Fisher Scientific, or BLDpharm and used without further purification. The stationary phase for column chromatography was 70–230 mesh silica 60 (Merck). The nuclear magnetic resonance (NMR) spectra were recorded on a Bruker Avance III HD 400 or a Bruker Avance II+ 600. ^13^C{^1^H} NMR spectra were acquired using power‐gated broad‐band decoupling. The chemical shifts (δ, ppm) were determined relative to the internal solvent signal. Coupling constants (*J*) are reported in Hertz (Hz) and were directly obtained from the spectra. The following abbreviations were used: s (singlet), d (doublet), t (triplet), and m (multiplet) to indicate the multiplicity of the peaks. The high‐resolution mass spectra (HRMS) were acquired on a quadrupole orthogonal acceleration time‐of‐flight mass spectrometer (Synapt G2 HDMS, Waters, Milford, MA). Samples were infused at 3 μL min^−1^ and the spectrum was obtained in positive ionization mode with a resolution of 15,000 (FWHM) using leucine enkephalin as lock mass. Melting points were determined on a Reichert‐Jung Thermovar system and are uncorrected.

### 4.2 Synthesis of Starting Materials

#### 4.2.1 *N*,*N*′‐(Ethane‐1,2‐diyl)bis(4‐methylbenzenesulfonamide) (1)

Compound **1** was synthesized using a reported method [3]. In a round‐bottom flask, ethylenediamine (2.20 mL, 33.28 mmol) was dissolved in 140 mL of pyridine at 0°C and *p*‐toluene sulfonyl chloride (13.00 g, 68.22 mmol) was added. After stirring at room temperature for 18 h, the mixture was poured into 400 mL of water. The resulting precipitate was filtered, washed 3x with 40 mL of diethyl ether, and dried in vacuo to obtain the title compound as a white powder in a yield of 95% (11.67 g). ^1^ **H NMR** (400 MHz, chloroform‐*d*) δ 7.71 (d, *J* = 8.2 Hz, 4H), 7.31 (d, *J* = 8.0 Hz, 4H), 4.85 (s, 2H), 3.06 (d, *J* = 6.0 Hz, 4H), 2.43 (s, 6H). ^1^H NMR data were in accordance with the data reported in the literature [27].

### 4.3 Macrocyclization

#### 4.3.1 Procedure A: Including Isolation of Intermediate 6

##### 4.3.1.1 *N*,*N*′‐(Ethane‐1,2‐diyl)bis(*N*‐(2‐(2‐chloroethoxy)ethyl)‐4‐methylbenzenesulfonamide) (6)

In a flame‐dried Ar‐flushed reaction tube, bis(2‐chloroethyl) ether **2** (25.00 mL, 232.50 mmol), Cs_2_CO_3_ (11.05 g, 33.92 mmol), and *N*,*N*′‐(ethane‐1,2‐diyl)bis(4‐methylbenzenesulfonamide) **1** (5.00 g, 13.57 mmol) were dissolved in 25 mL of dry ACN. The reaction mixture was stirred at 80°C for 18 h. The reaction was monitored via TLC and after full conversion of the starting material the reaction mixture was cooled down to room temperature. Next, the undissolved Cs_2_CO_3_ was removed by centrifugation and washed once with 40 mL of EtOAc. The solvents were combined and removed at the rotavapor. Afterwards, 200 mL of cyclohexane was added, and the mixture was left overnight in the fridge, resulting in the formation of clear crystals and a brown oil. Most of the crystals were collected by filtration using a Büchner filter and washed with 100 mL of cyclohexane to remove the excess bis(2‐chloroethyl) ether **2**. A small fraction of the crystals, along with the brown oil, remained in the flask. The remaining crystals and brown oil were combined with the filtered crystals, redissolved in EtOAc, and coated onto Celite for further purification. Next, column chromatography was performed using petroleum ether/ EtOAc 3:1 to obtain the product as a white solid in a yield of 62% (4.92 g). **MP**: 82°C–84°C. **HRMS** (ESI‐Q‐TOF): m/z [M + H]^+^ calcd. for C_24_H_34_Cl_2_N_2_O_6_S_2_: 581.1308; found: 581.1313. ^1^ **H NMR** (400 MHz, chloroform‐*d*) δ 7.71 (d, *J* = 8.3 Hz, 4H), 7.31 (d, *J* = 7.9 Hz, 4H), 3.70–3.60 (m, 8H), 3.59–3.49 (m, 4H), 3.41 (s, 4H), 3.34 (t, *J* = 5.5 Hz, 4H), 2.43 (s, 6H). ^13^ **C{** ^1^ **H} NMR** (101 MHz, chloroform‐*d*) δ 143.6, 136.1, 129.8, 127.3, 71.1, 70.1, 49.5, 49.2, 42.7, 21.5.

##### 4.3.1.2 4,7‐Ditosyl‐1‐oxa‐4,7‐diazacyclononane (3)

Compound **3** was isolated by chromatographic separation in 4 % yield as a byproduct in the synthesis of compound **6**. **MP**: 193°C–195°C. **HRMS** (ESI‐Q‐TOF): m/z [M + H]^+^ calcd. for C_20_H_26_N_2_O_5_S_2_: 439.1356; found: 439.1357. ^1^ **H NMR** (600 MHz, chloroform‐*d*) δ 7.69 (d, *J* = 8.2 Hz, 4H), 7.32 (d, *J* = 8.0 Hz, 4H), 3.90 (t, *J* = 4.2 Hz, 4H), 3.46 (s, 4H), 3.26 (t, *J* = 4.2 Hz, 4H), 2.43 (s, 6H). ^13^ **C{** ^1^ **H} NMR** (101 MHz, chloroform‐*d*) δ 143.7, 135.2, 129.9, 127.3, 72.0, 52.1, 51.9, 21.5. ^1^H NMR data were in accordance with the data reported in the literature [6].

##### 4.3.1.3 4,7,13,16‐Tetratosyl‐1,10‐dioxa‐4,7,13,16‐tetraazacyclooctadecane (4)

In a flame‐dried Ar‐flushed reaction tube, *N*,*N*′‐(ethane‐1,2‐diyl)bis(*N*‐(2‐(2‐chloroethoxy)ethyl)‐4‐methylbenzenesulfonamide) **6** (4.2 g, 7.22 mmol), K_2_CO_3_ (4.99 g, 36.11 mmol), and *N*,*N*′‐(ethane‐1,2‐diyl)bis(4‐methylbenzenesulfonamide) **1** (2.93 g, 7.94 mmol) were dissolved in 420 mL of dry DMF. The reaction mixture was stirred for 18 h at 110°C. Thereafter, the DMF was evaporated until ±100 mL was left and 500 mL of water was added resulting in the formation of a white precipitate. This precipitate was filtered over a Buchner filter and washed 3× with 50 mL of water. The product was dried in a vacuum oven at 50°C resulting in a yield of 80% (5.09 g). Melting point and HRMS data were in accordance with the data reported in the literature [3, 14]. **MP**: 241°C–243°C. **HRMS** (ESI‐Q‐TOF): m/z [M + H]^+^ calcd. for C_40_H_52_N_4_O_2_S_4_: 877.2639; found: 877.2616. ^1^ **H NMR** (400 MHz, chloroform‐*d*) δ 7.71 (d, *J* = 8.3 Hz, 8H), 7.32 (d, *J* = 8.1 Hz, 8H), 3.54 (t, *J* = 5.0 Hz, 8H), 3.32 (s, 8H), 3.22 (t, *J* = 5.0 Hz, 8H), 2.44 (s, 12H). ^13^ **C{** ^1^ **H} NMR** (101 MHz, chloroform‐*d*) δ 143.6, 135.8, 129.9, 127.3, 71.7, 50.6, 50.2, 21.6.

#### 4.3.2 Procedure B: Without Isolation of Intermediate 6

##### 4.3.2.1 *N*,*N*′‐(Ethane‐1,2‐diyl)bis(*N*‐(2‐(2‐chloroethoxy)ethyl)‐4‐methylbenzenesulfonamide) (Mixture of 6 and 3)

In a flame‐dried Ar‐flushed reaction tube, bis(2‐chloroethyl) ether **2** (12.50 mL, 116.25 mmol), Cs_2_CO_3_ (5.53 g, 16.96 mmol), and *N*,*N*′‐(ethane‐1,2‐diyl)bis(4‐methylbenzenesulfonamide) **1** (2.50 g, 6.79 mmol) were dissolved in 12.5 mL of dry ACN. The reaction mixture was stirred at 80°C for 18 h. The reaction was monitored via TLC, and after full conversion of the starting material, the reaction mixture was cooled down to room temperature. Next, the undissolved Cs_2_CO_3_ was removed by centrifugation and washed 1× with 20 mL of EtOAc. The solvents were removed at the rotavapor. Afterwards, 100 mL of cyclohexane was added and the mixture was left overnight in the fridge, resulting in the formation of clear crystals and a brown oil. Next, the cyclohexane fraction is decanted. Another 20 mL of cyclohexane was added to the residue, and the mixture was sonicated. Afterwards, the cyclohexane was decanted. This washing procedure was repeated three times to completely remove bis(2‐chloroethyl) ether **2**, followed by thorough drying at the Schlenck line. The crude product (2.17 g) was obtained as a brown solid and used in the next step without further purification.

##### 4.3.2.2 4,7,13,16‐Tetratosyl‐1,10‐dioxa‐4,7,13,16‐tetraazacyclooctadecane (4)

In a flame‐dried Ar‐flushed reaction tube, *N*,*N*′‐(ethane‐1,2‐diyl)bis(4‐methylbenzenesulfonamide) **1** (1.35 g, 3.67 mmol), K_2_CO_3_ (2.30 g, 16.68 mmol), and the precipitated mixture of **6** and **3** (2.17 g) were dissolved in 194 mL of dry DMF. The reaction mixture was stirred for 18 h at 110°C. Thereafter, the DMF was evaporated until ±50 mL was left and 100 mL of water was added resulting in the formation of a sticky off‐white precipitate. The solvent (H_2_O/DMF) was decanted and 20 mL of acetone was added. This mixture was sonicated resulting in the formation of a white precipitate. The solid was filtered over a Büchner filter and washed with 10 mL of acetone. After drying at the Schlenck line, the product was obtained as a white solid in a yield of 28% over two steps (1.64 g). Characterization data are mentioned under Procedure A.

### 4.4 Tosyl Deprotection

#### 4.4.1 1,10‐Dioxa‐4,7,13,16‐tetraazacyclooctadecane (5)

In a round‐bottom flask, 4,7,13,16‐tetratosyl‐1,10‐dioxa‐4,7,13,16‐tetraazacyclooctadecane **4** (5.00 g, 5.70 mmol) was dissolved in 50 mL of concentrated H_2_SO_4_ and heated at 100°C for 7 h. Afterwards, the mixture was placed in an ice bath and a sodium hydroxide solution (6 M) was added carefully to the reaction mixture to adjust the pH to 14. Next, the mixture was extracted with chloroform (10 × 100 mL), dried with Na_2_SO_4_, and evaporated to obtain the product as an off‐white solid in a yield of 72% (1.08 g). Characterization data were in accordance with the data reported in the literature [3, 14]. **MP**: 59°C–61°C. **HRMS** (ESI‐Q‐TOF): m/z [M + H]^+^ calcd. for C_12_H_28_N_4_O_2_: 261.2285; found: 261.2285. ^1^ **H NMR** (400 MHz, DMSO‐*d*_6_) δ 3.44 (t, *J* = 4.6 Hz, 8H), 2.63 (t, *J* = 4.7 Hz, 8H), 2.59 (s, 8H). ^13^ **C{** ^1^ **H} NMR** (101 MHz, DMSO‐*d*_6_) δ 69.7, 49.0, 48.9.

## Supporting Information

Additional supporting information can be found online in the Supporting Information section.

## Funding

This work was funded by KU Leuven (C14/19/78).

## Conflicts of Interest

The authors declare no conflicts of interest.

## Supporting information

Supplementary Material

## Data Availability

The alternative synthetic pathway to TA18C6, synthetic procedures, and characterization data for compounds **7**–**9** and NMR spectra are available in the Electronic Supplementary Information (ESI) associated with this article.
