# Supplementary material for: A Stepwise Approach to 1,4,10,13‐Tetraaza‐18‐Crown‐6 Ether
Source: ChemistryOpen. 2026 May 5;15(5):e70201. doi: 10.1002/open.70201 (PMC13140966; doi:10.1002/open.70201)
Supplement: Supplementary file 1 — Supplementary Material [file OPEN-15-e70201-s001.pdf]

Electronic Supplementary Information (ESI)

A stepwise approach to 1,4,10,13-tetraaza-18-crown-6 ether

P. Troosters[a,b], L. Bruneel[a,b], W. Dehaen*[b], T. Opsomer*[c]

[a.] Nuclear Energy Technology, Belgian Nuclear Research Centre (SCK CEN), B-2400 Mol, Belgium
[b.] Department of Chemistry, KU Leuven, B-3001 Leuven, Belgium
[c.] Nuclear Medical Applications, Belgian Nuclear Research Centre (SCK CEN), B-2400 Mol, Belgium

* E-mail: tomas.opsomer@sckcen.be, wim.dehaen@kuleuven.be

Table of Contents

Alternative synthetic pathway to TA18C6 3

Synthetic procedures and characterization data 4

NMR spectra (^1H NMR and ^{13}C NMR) 5

Calculating the yield of intermediate 6 in the precipitated mixture of 6 and 3 12

Alternative synthetic pathway to TA18C6

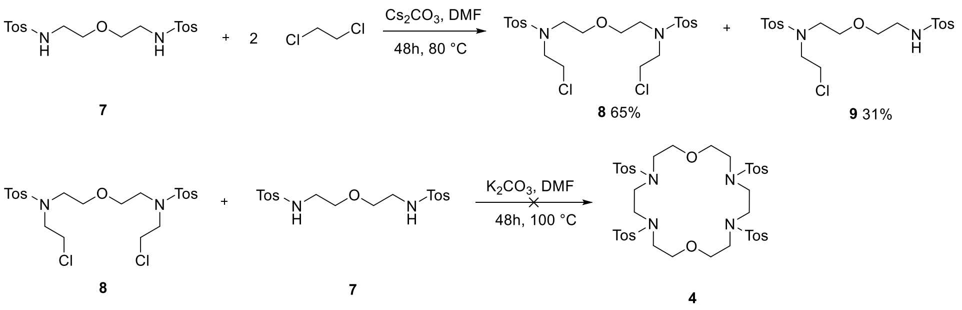

Scheme S1 Alternative synthetic pathway to TA18C6. Full conversion to compound **8** was not achieved in this reaction, resulting in a yield of intermediate **9** of 31 %. The macrocyclization towards tetratosyl-TA18C6 **4** was unsuccessful, partly due to β-elimination in compound **8** as a side reaction, which also contributed to purification difficulties.

Table S1 Different reaction conditions tested for the reaction of *N,N'*-(oxybis(ethane-2,1-diyl))bis(4-methylbenzenesulfonamide) **7** and DCE.[a]

Entry	Base/equiv.	Solvent/V (mL)	Time (h)	V DCE (mL)/equiv.	Yield 8 (%)[b]	Yield 9 (%)[b]
1[c]	Cs_2CO_3/2.5	ACN/0.5	18	1.5/78	34	38
2	Cs_2CO_3/2.5	ACN/0.5	18	1.0/52	48	37
3[d]	Cs_2CO_3/2.5	ACN/0.5	18	0.5/26	40	38
4	Cs_2CO_3/2.5	ACN/0.5	48	1.0/52	42	31
5	Cs_2CO_3/2.5	DMF/0.5	48	1.0/52	46	38
6	Cs_2CO_3/2.5	DMF/0.3	48	1.0/52	65	31
7	Cs_2CO_3/2.5	DMF/0.3	60	1.0/52	63	32

[a] The reaction temperature was 80 °C. [b] Isolated yields. [c] 5 % of starting material **7** was still present. [d] 4 % of starting material **7** was still present.

Different reaction conditions were evaluated to synthesize compound **8** as visualized in Table S1. TLC analysis was used to monitor the reaction, revealing that full conversion of starting material **7** to product **8** was never achieved. In contrast to the other route presented in the manuscript, the 9-membered ring was not observed. For the optimization, the amount of DCE was reduced from 78 to 26 equivalents (Entry 1-3). Using 52 equivalents of DCE (Entry 2) resulted in the highest yield for product **8**, with full conversion of the starting material **7** to **8** and **9**. Next, the reaction time was increased to 48 h (Entry 4). Unfortunately, this did not result in a higher yield of **8**. Subsequently, DMF was evaluated as an alternative solvent to ACN, which led to only a slight increase in the yield of product **8** (Entries 4 and 5). A significant improvement was observed when the solvent volume was reduced to 0.3 mL, resulting in a yield of 65 % (Entry 6). This suggested that more concentrated conditions are beneficial for this reaction. Finally, the reaction time was increased to 60 h, which did not result in higher conversion (Entry 7). To summarize, the highest yield for product **8** was obtained in entry 6, using 0.3 mL of DMF as solvent with 52 equivalents of DCE and a reaction time of 48 h.

Synthetic procedures and characterization data

_N,N'_-(oxybis(ethane-2,1-diyl))bis(4-methylbenzenesulfonamide) (7)

Compound **7** was synthesized using a reported method.[26] 2,2′-Oxybis(ethan-1-amine) (1.00 g, 9.60 mmol) was dissolved in dry ACN (45 mL), Et_3N (8 mL, 0.06 mmol) was added, and the resulting solution was cooled in ice. To this solution, *p*-toluenesulfonyl chloride (3.75 g, 19.68 mmol) was added in small portions. The mixture was then heated to 65 °C and left stirring for 22 h. The organic solvent was removed *in vacuo* and replaced with DCM (100 mL). An extraction was performed with 1 M HCl, and the organic layer was concentrated to dryness. The product was recrystallized in 30 mL DCM and washed with diethyl ether to obtain the product as a white solid in a yield of 79% (3.14 g). **^{1}H NMR** (600 MHz, chloroform-*d*) δ 7.75 (d, *J* = 8.2 Hz, 4H), 7.31 (d, *J* = 8.2 Hz, 4H), 5.12 (t, *J* = 6.1 Hz, 2H), 3.37 (t, *J* = 5.0 Hz, 4H), 3.08 (q, *J* = 5.6 Hz, 4H), 2.43 (s, 6H). Characterization data were in accordance with the data reported in literature.[26]

_N,N'_-(oxybis(ethane-2,1-diyl))bis(_N_-(2-chloroethyl)-4-methylbenzenesulfonamide) (8)

In a flame-dried Ar-flushed reaction tube, dry 1,2-dichloroethane (1.00 mL, 12.66 mmol), Cs_2CO_3 (0.17 g, 0.53 mmol) and *N,N'*-(oxybis(ethane-2,1-diyl))bis(4-methylbenzenesulfonamide) (0.10 g, 0.24 mmol) were dissolved in 0.3 ml of dry DMF. The reaction mixture was stirred at 80 °C for 48 h. Next, the undissolved Cs_2CO_3 was removed by centrifugation and washed 1x with 5 mL of EtOAc. The solvents were combined and removed at the rotavapor. The remaining solid was dissolved in EtOAc and coated onto Celite for further purification. Column chromatography was performed using 25% EtOAc/petroleum ether to obtain the product as a white solid in a yield of 65 % (85.0 mg). **MP**: 84-86°C. **HRMS** (ESI-Q-TOF): m/z $[M + H]^+$ calcd. for $C_{22}H_{30}Cl_2N_2O_5S_2$: 537.1046; found: 537.1050. **^{1}H NMR** (400 MHz, chloroform-*d*) δ 7.69 (d, *J* = 8.3 Hz, 4H), 7.32 (d, *J* = 8.0 Hz, 4H), 3.64 (t, *J* = 7.4 Hz, 4H), 3.58 (t, *J* = 5.5 Hz, 4H), 3.42 (t, *J* = 7.4 Hz, 4H), 3.32 (t, *J* = 5.5 Hz, 4H), 2.43 (s, 6H). **^{13}C{^{1}H} NMR** (151 MHz, chloroform-*d*) δ 143.8, 136.1, 129.9, 127.2, 70.6, 51.4, 49.4, 41.9, 21.6.

_N_-(2-chloroethyl)-4-methyl-_N_-(2-(2-(4-methylphenylsulfonamido)ethoxy)ethyl)benzenesulfonamide (9)

Compound **9** was obtained in a yield of 31 % as a byproduct in the synthesis of compound **8**. **MP**: 64-66 °C. **HRMS** (ESI-Q-TOF): m/z $[M + H]^+$ calcd. for $C_{20}H_{27}Cl_1N_2O_5S_2$: 475.1123; found: 475.1120. **^{1}H NMR** (600 MHz, chloroform-*d*) δ 7.74 (d, *J* = 8.0 Hz, 2H), 7.68 (d, *J* = 8.0 Hz, 2H), 7.33 – 7.28 (m, 4H), 5.03 (t, *J* = 5.7 Hz, 1H), 3.58 (t, *J* = 7.2 Hz, 2H), 3.51 (t, *J* = 5.2 Hz, 2H), 3.43 (t, *J* = 4.9 Hz, 2H), 3.38 (t, *J* = 7.2 Hz, 2H), 3.31 (t, *J* = 5.2 Hz, 2H), 3.08 (q, *J* = 5.2 Hz, 2H), 2.42 (s, 3H), 2.41 (s, 3H). **^{13}C{^{1}H} NMR** (151 MHz, chloroform-*d*) δ 143.9, 143.5, 136.9, 136.0, 129.9, 129.8, 127.2, 127.1, 70.0, 69.4, 51.1, 49.2, 42.9, 41.9, 21.6, 21.5.

NMR spectra (^{1}H NMR and ^{13}C NMR)

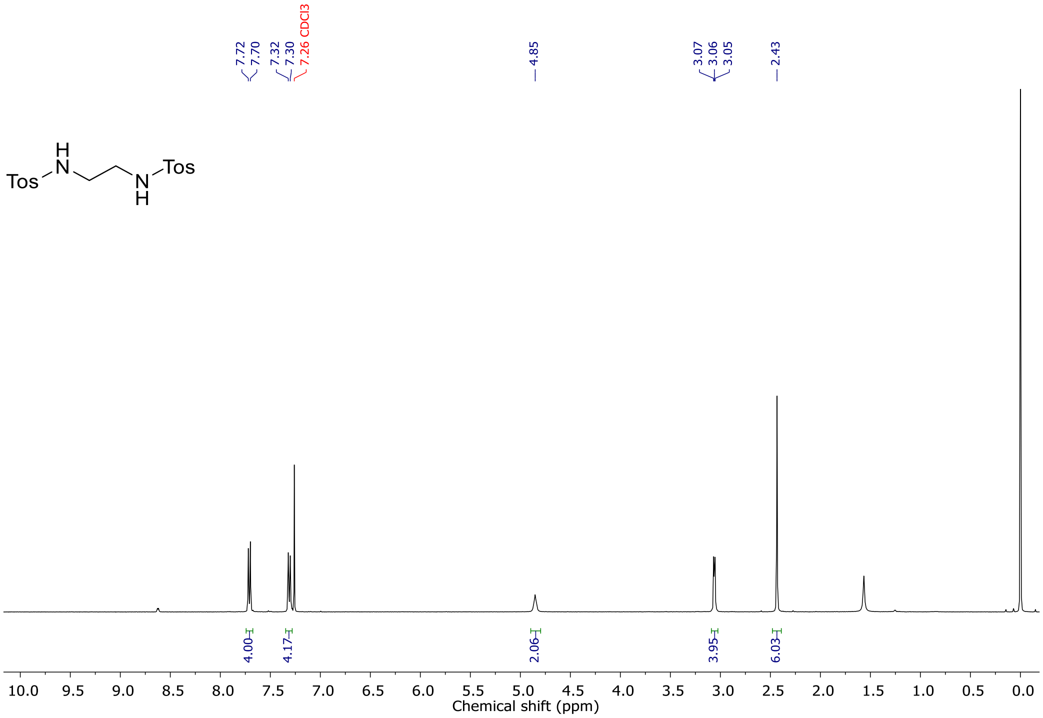

*Figure S1: ^{1}H NMR spectrum of compound **1**.*

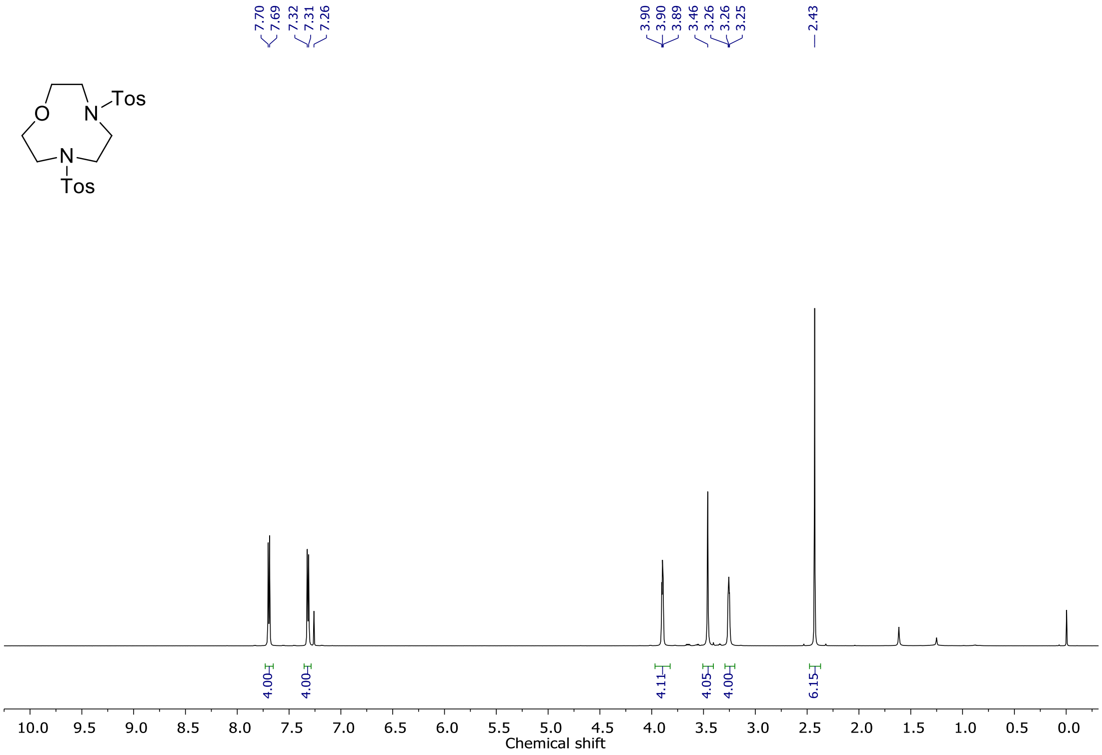

*Figure S2: ^{1}H NMR spectrum of compound **3**.*

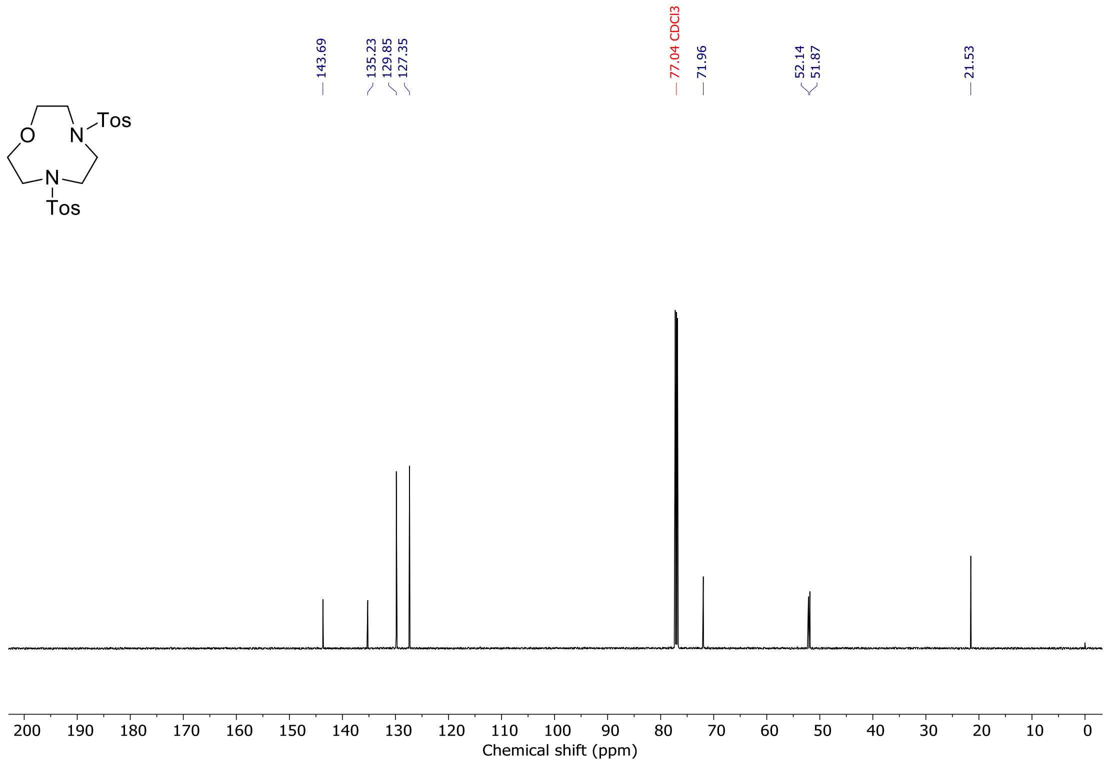

*Figure S3: ^{13}C NMR spectrum of compound **3**.*

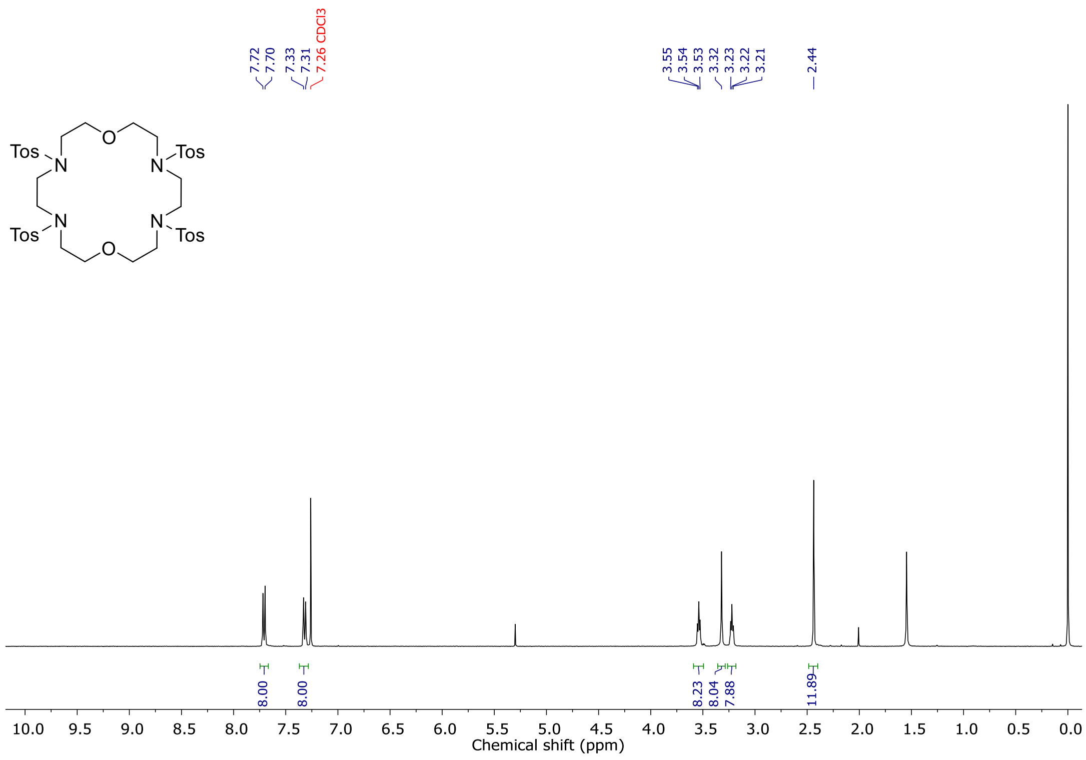

*Figure S4: ^{1}H NMR spectrum of compound **4**.*

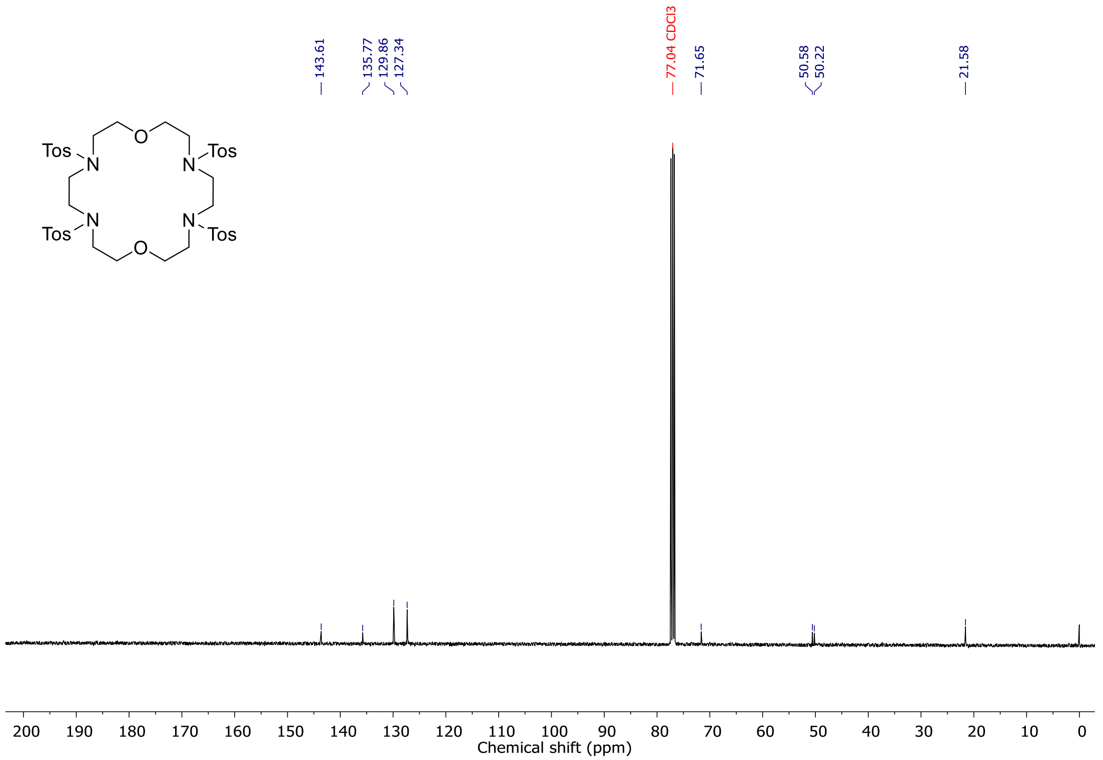

*Figure S5: ^{13}C NMR spectrum of compound **4**.*

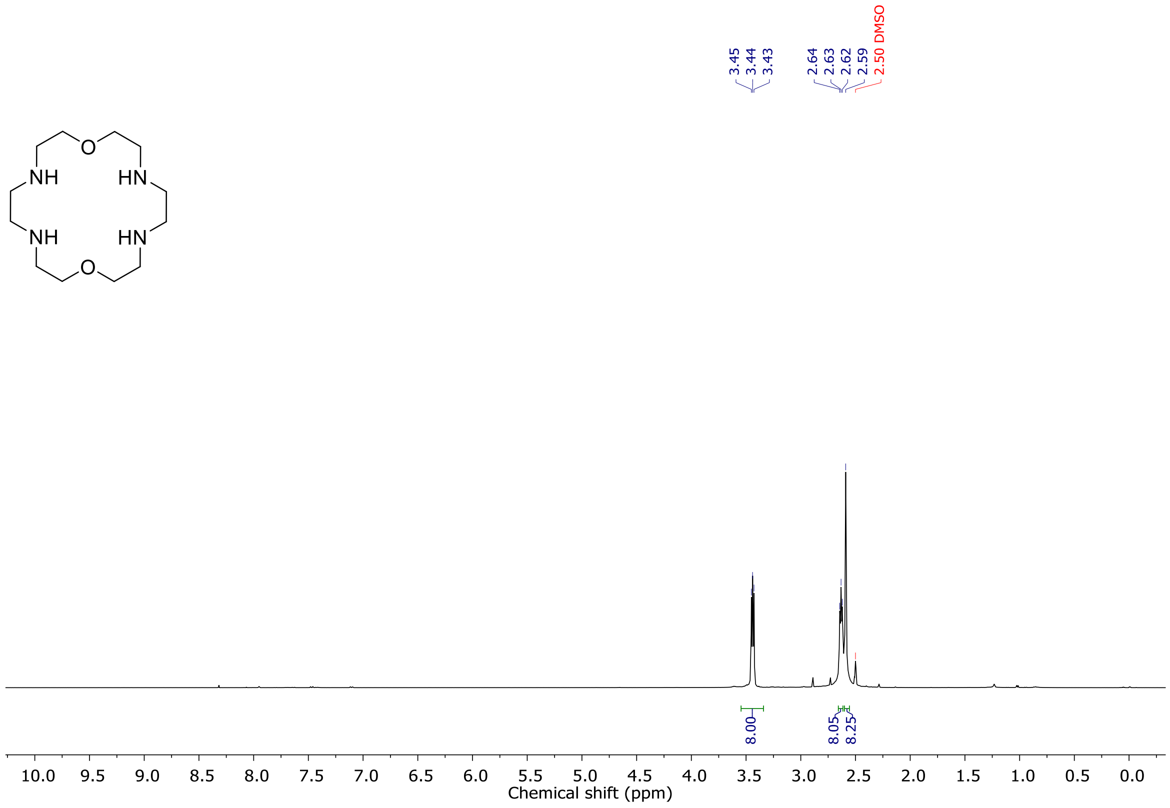

*Figure S6: ^{1}H NMR spectrum of compound **5**.*

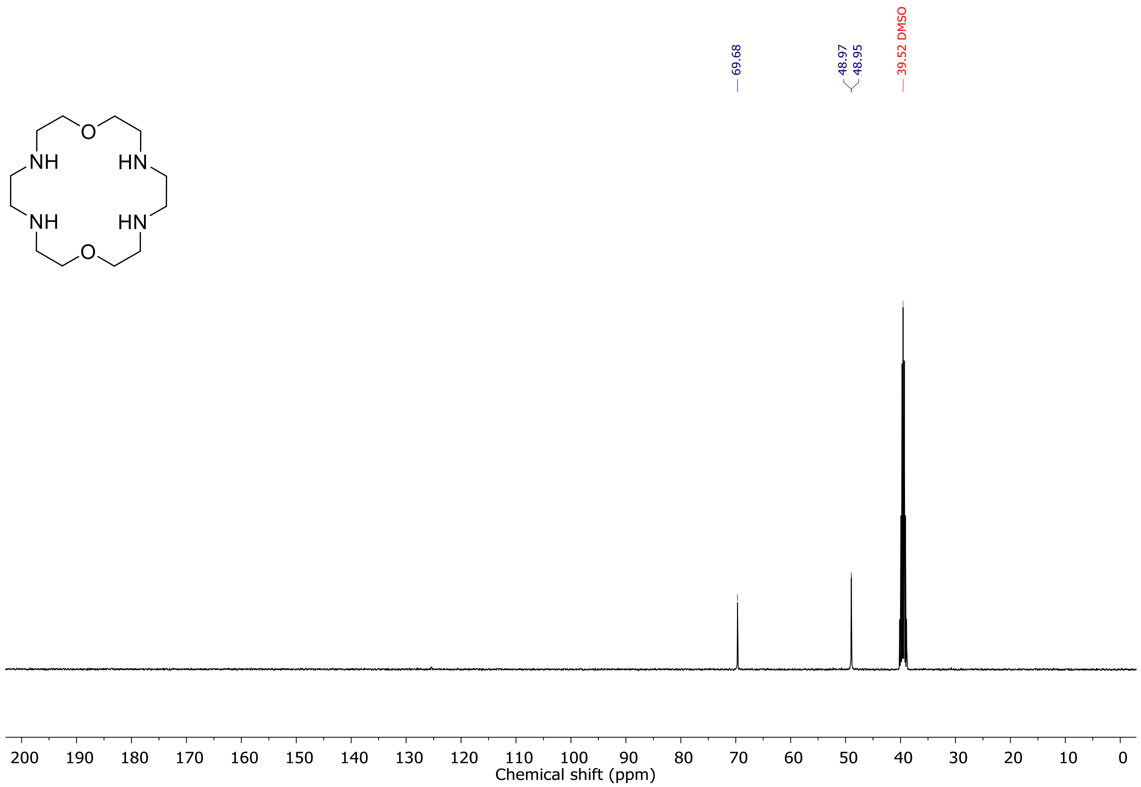

*Figure S7: ^{13}C NMR spectrum of compound **5**.*

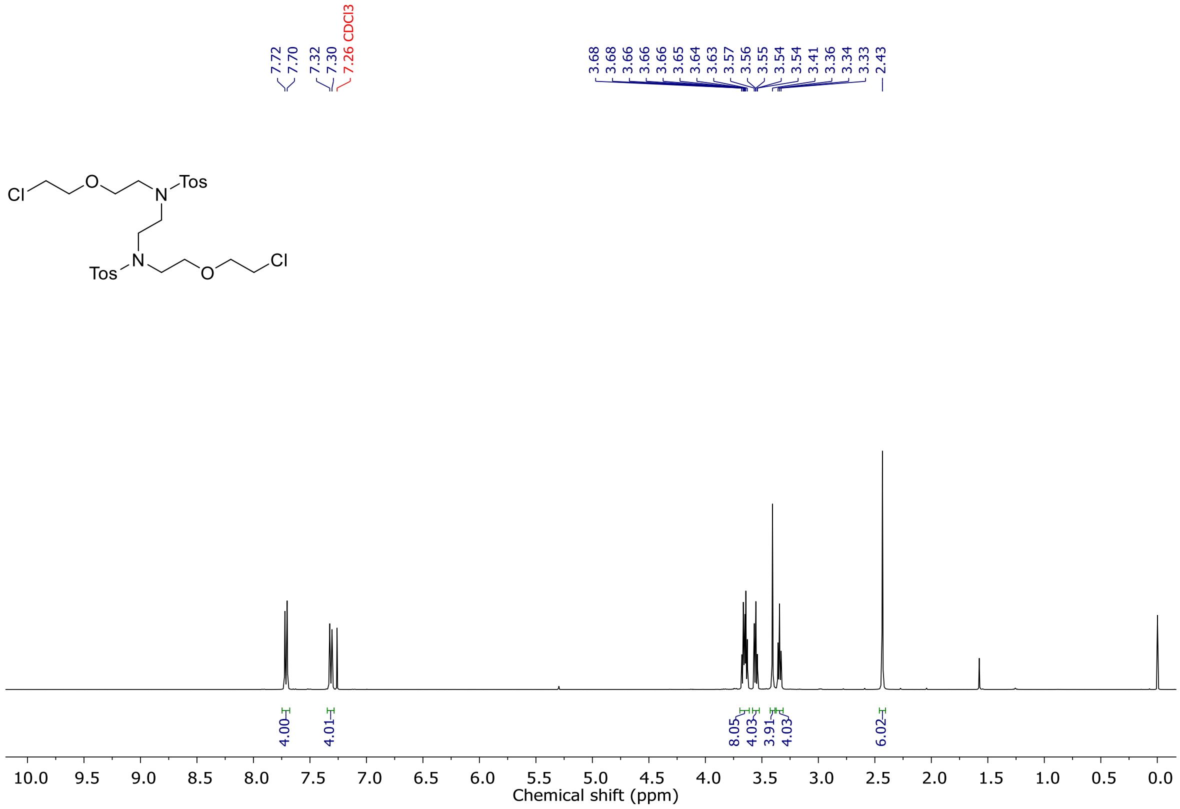

*Figure S8: ^{1}H NMR spectrum of compound **6**.*

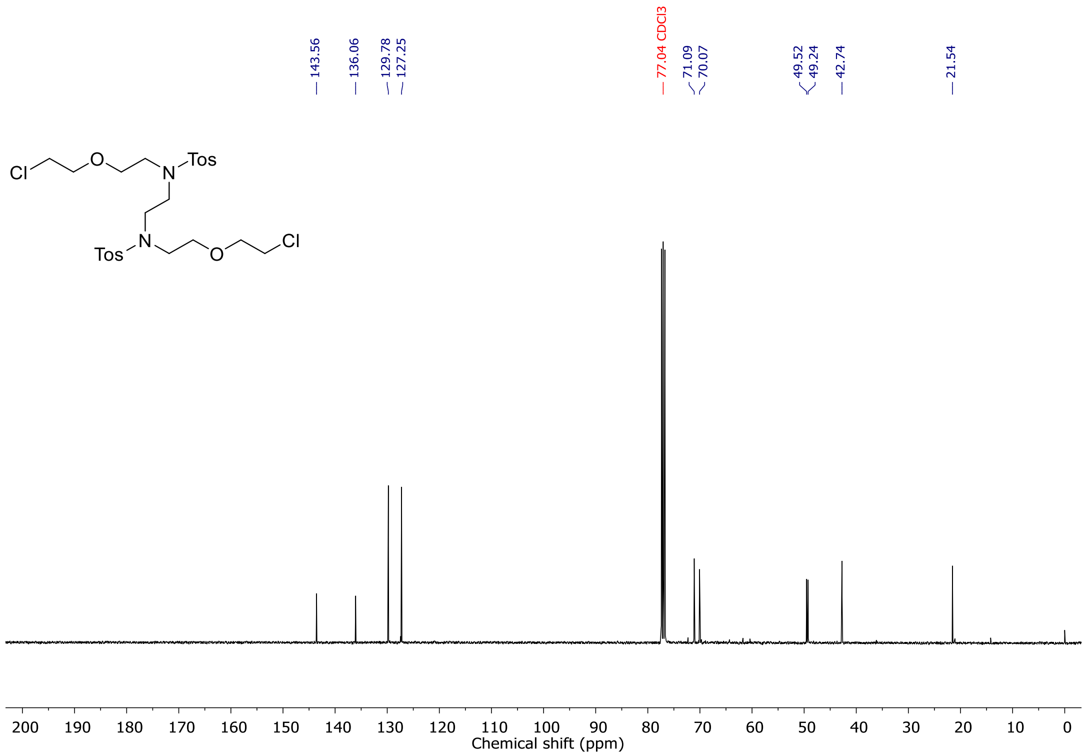

*Figure S9: ^{13}C NMR spectrum of compound **6**.*

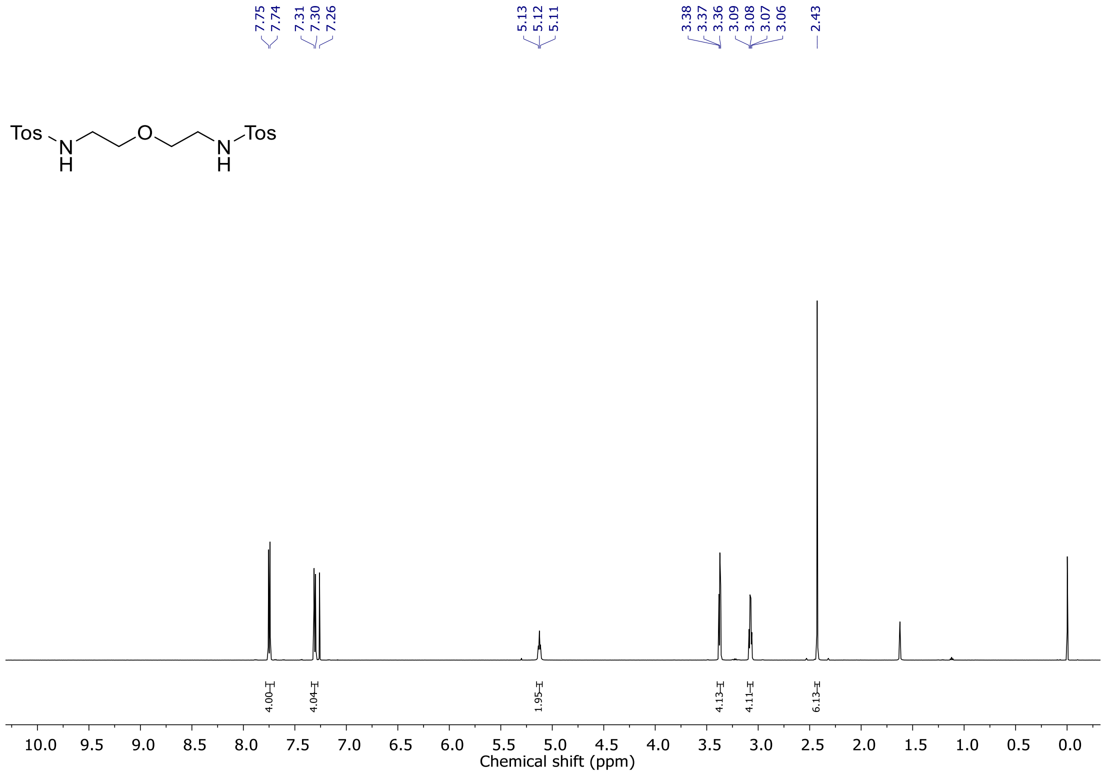

*Figure S10: ^{1}H NMR spectrum of compound **7**.*

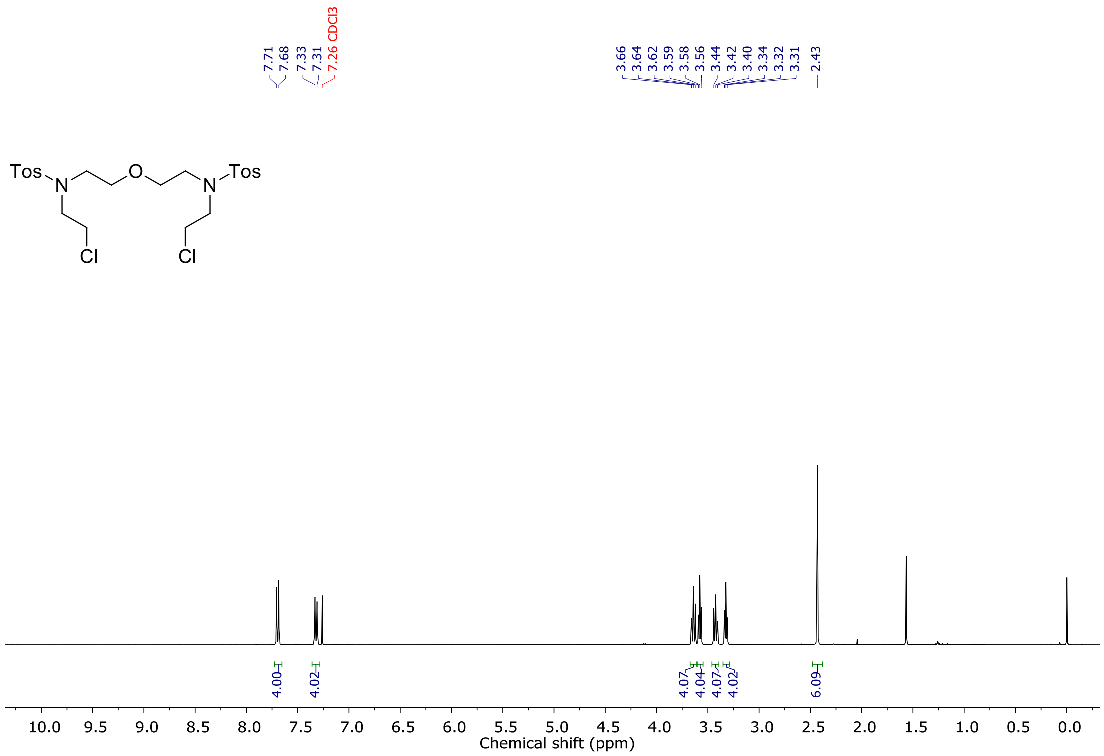

*Figure S11: ¹H NMR spectrum of compound **8**.*

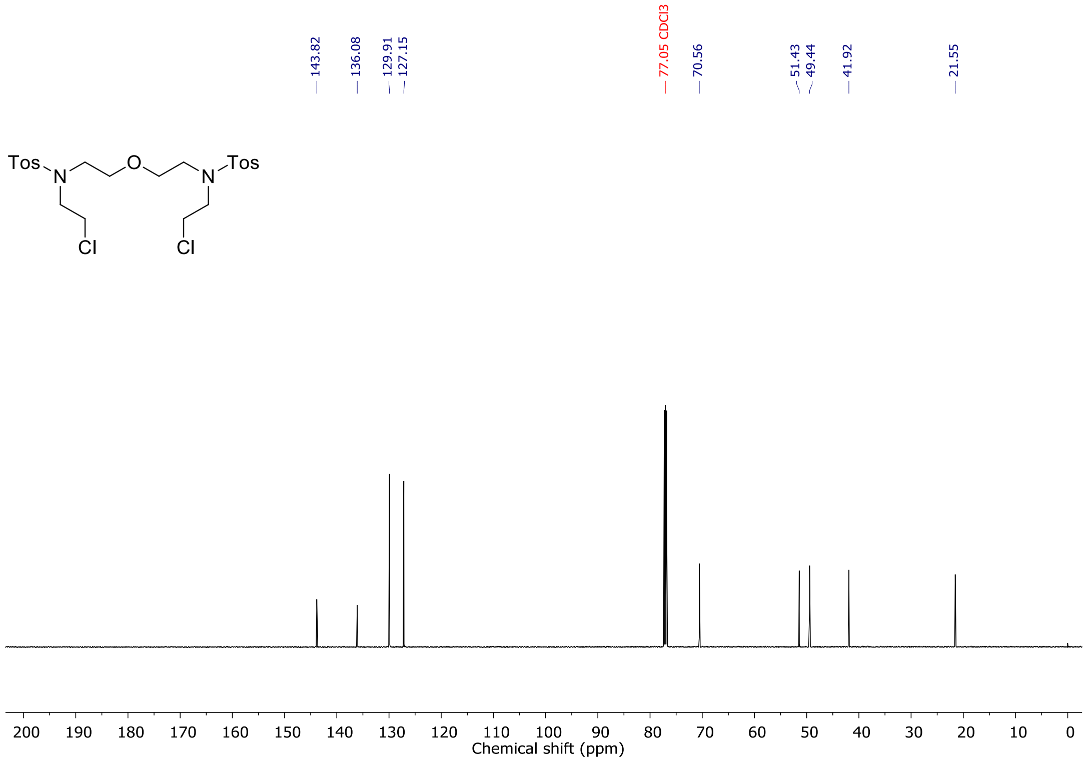

*Figure S12: ¹³C NMR spectrum of compound **8**.*

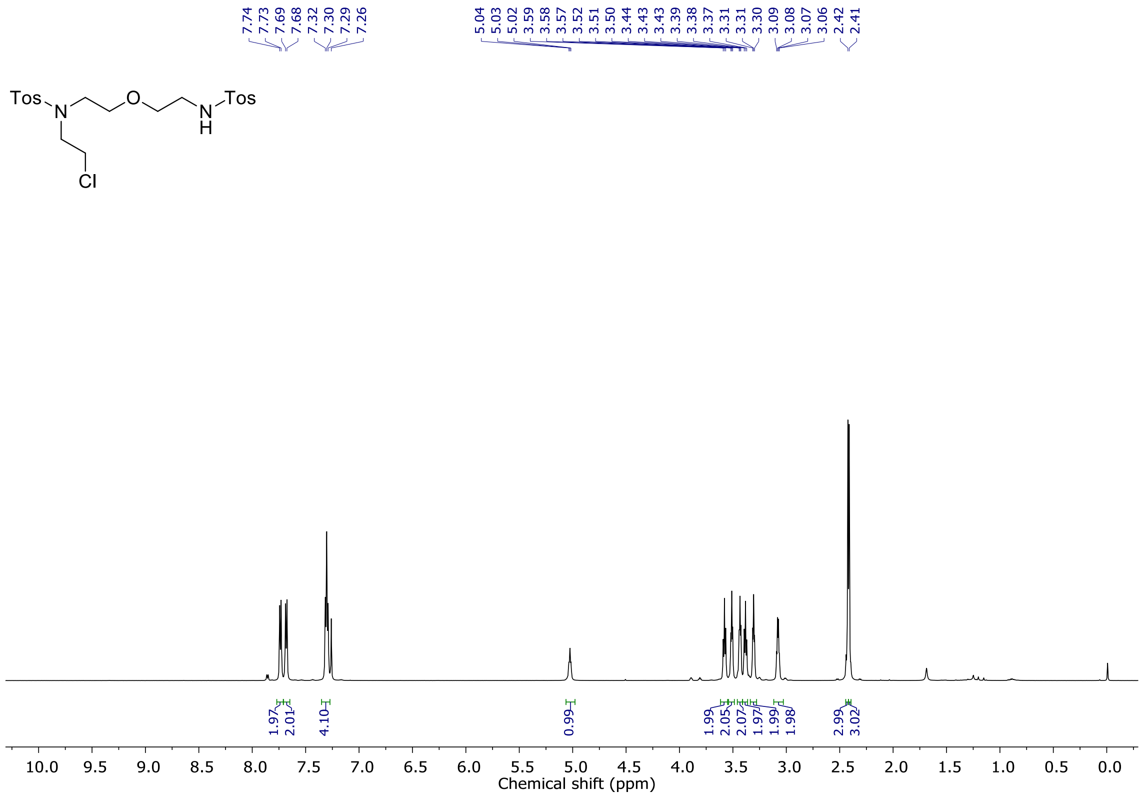

*Figure S13: ¹H NMR spectrum of compound **9**.*

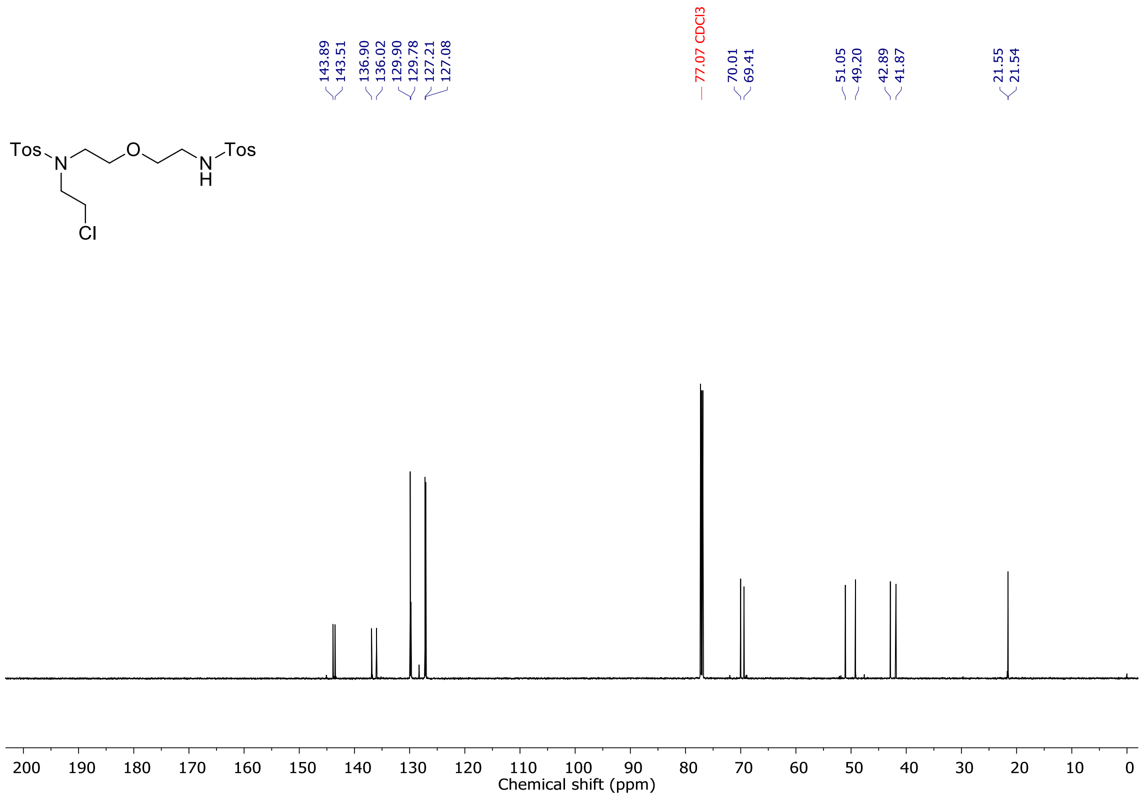

*Figure S14: ¹³C NMR spectrum of compound **9**.*

Calculating the yield of intermediate 6 in the precipitated mixture of 6 and 3

After coprecipitation of intermediate **6** and side product **3**, the mixture was repeatedly washed with cyclohexane. This mixture was analyzed with ^{1}H NMR spectroscopy to determine the ratio between intermediate **6** and side product **3** (Figure S15). The mixture has a total mass of 2.17 g and the molecular weights (MW) of **6** and **3** are 581.56 g/mol and 438.56 g/mol, respectively. According to the ^{1}H NMR spectrum (Figure S15), the mole fraction of **6** was $\frac{1}{1+0.1} = 0.91$ and the mole fraction of **3** was $\frac{0.1}{1+0.1} = 0.09$. Of the starting ditosylethylenediamine **1**, 6.79 mmol was used.

The yield of **6** was calculated as follows:

$$[MW\ (\mathbf{6}) * mole\ fraction\ (\mathbf{6}) + MW\ (\mathbf{3}) * mole\ fraction\ (\mathbf{3})] * n(\mathbf{6}+\mathbf{3}) = m\ (\mathbf{6}+\mathbf{3})$$

$$n(\mathbf{6}+\mathbf{3}) = \frac{m\ (\mathbf{6}+\mathbf{3})}{MW\ (\mathbf{6}) * mole\ fraction\ (\mathbf{6}) + MW\ (\mathbf{3}) * mole\ fraction\ (\mathbf{3})}$$

$$n(\mathbf{6}+\mathbf{3}) = \frac{2.17\ g}{581.56 \frac{g}{mol} * 0.91 + 438.56 \frac{g}{mol} * 0.09} = 0.003816\ mol$$

$$n(\mathbf{6}) = mole\ fraction\ (\mathbf{6}) * n(\mathbf{6}+\mathbf{3})$$

$$n(\mathbf{6}) = 0.91 * 0.003816\ mol = 0.00347\ mol$$

$$Yield\ (\mathbf{6}) = \frac{3.47\ mmol}{6.79\ mmol} = 51\ \%$$

The yield of **6** is 51 %, which is 11 % lower compared to the 62 % obtained when **6** was purified *via* column chromatography. Based on these calculations, it can be concluded that approximately 11 % of the product is lost due to the additional cyclohexane washing steps.

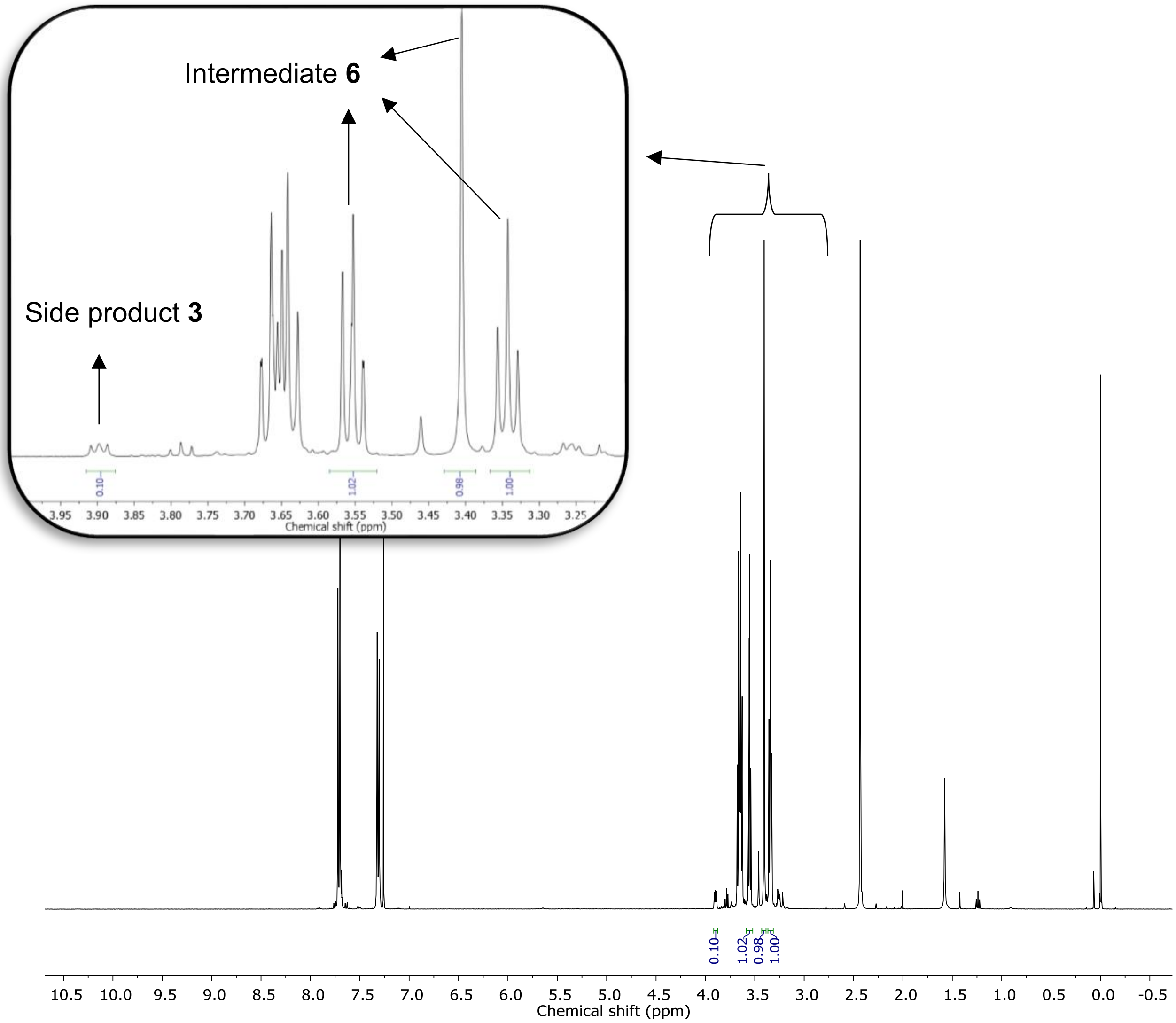

*Figure S15: ^{1}H NMR spectrum of the mixture of intermediate **6** and side product **3** after cyclohexane washings.*
